# The influence of diverse bone cement distribution patterns for metastatic vertebral lesions after bilateral percutaneous kyphoplasty

**DOI:** 10.1186/s12891-022-05680-4

**Published:** 2022-07-26

**Authors:** Wence Wu, Xinxin Zhang, Xiaoyang Li, Shengji Yu

**Affiliations:** Department of Orthopedics, National Cancer Center/National Clinical Research Center for Cancer/Cancer Hospital, Chinese Academy of Medical Sciences and Peking Union Medical College, Beijing, People’s Republic of China

**Keywords:** Confluent, Separated, Kyphoplasty, Spinal metastases, Pain

## Abstract

**Objective:**

To investigate the influence of diverse bone cement distribution patterns in patients with metastatic vertebral lesions after bilateral percutaneous kyphoplasty (PKP).

**Methods:**

Fifty-nine patients with single-level metastatic vertebral lesions who received bilateral PKP were retrospectively reviewed. According to the different bone cement distribution patterns, patients were divided into confluent (*n* = 35, CF) and separated (*n* = 24, SP) groups. Indicators including visual analogue scale (VAS), Oswestry Disability Index (ODI), vertebral body height (VBH) variation, quality of life (QoL), and related complications were reviewed and compared between the two groups.

**Results:**

No statistically significant differences were observed between the two groups in age, sex, types of lesions, locations of lesions, posterior vertebral body and/or pedicle involvement, percentage of vertebral invasion, procedure duration or cement volume (*p* > 0.05). There was significant improvement in VAS, ODI, VBH and QoL at any follow-up examination (*p* < 0.05) compared with those preoperatively. The CF group exhibited better pain relief in VAS scores than did the SP group just at 3 days and 1 month after PKP (*p* < 0.05). There were no significant differences between the two groups in VAS scores at 3 months or 1 year after PKP (*p* > 0.05). No statistically significant differences were observed between the two groups in terms of ODI, VBH or QoL (*p* > 0.05). There was no statistically significant difference in the incidence of complications between the two groups (*p* > 0.05).

**Conclusions:**

More rapid pain relief was achieved with confluent rather than separated bone cement distribution patterns in PKP for patients with metastatic vertebral lesions.

## Introduction

Percutaneous kyphoplasty (PKP) has proven to be an effective treatment for spinal metastases with rapid pain relief and satisfactory results [1]. The distribution of bone cement in PKP is a key factor in maintaining vertebral stability and efficacy [2, 3]. However, most relevant studies have focused on the outcomes of the different distribution patterns of cement in osteoporotic vertebral compression fractures (OVCFs) [2, 4–6], and the involvement of bone cement distribution patterns in metastatic vertebral lesions has not yet been well reported. OVCFs usually lead to a loss of anterior VBH, while tumours usually invade the posterior vertebral body. Therefore, the cement distribution patterns in OVCFs may not be suitable for the management of metastatic vertebral lesions. As a result, this study aims to investigate the influence of diverse bone cement distribution patterns for patients with metastatic vertebral lesions after bilateral PKP.

## Materials and methods

### Inclusion and exclusion criteria

The inclusion criteria were as follows: (1) a definitive diagnosis of vertebral metastatic cancer; (2) an intact posterior margin of the vertebral body without neurological deficit, spinal cord compression, or canal narrowing; (3) survival time more than 3 months (according to the Tomita score); and (4) severe back pain that could not be alleviated by traditional cancer therapies.

Exclusion criteria: (1) primary bone tumours (e.g., osteosarcoma or osteoclastoma) or haematological disease (e.g., plasmacytoma); (2) severe cardiopulmonary disease, coagulation dysfunction or infections; (3) collapse of the posterior vertebral body wall and extension of the tumour into the spinal canal; (4) combined treatment of radiofrequency ablation (RFA) or fixateur interne with PKP; or (5) terminal patients or patient's refusal to undergo the procedure, or psychiatric disorders.

### Patients demographics

**Fig. 1:**
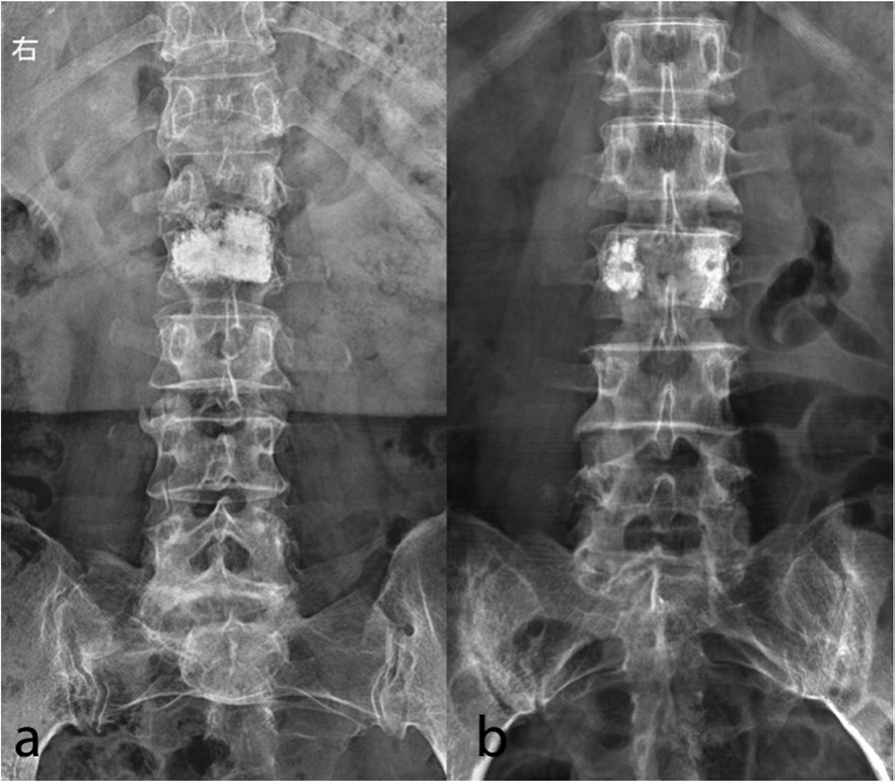
Postoperative frontal radiographs of the two bone cement distribution patterns. **a** Confluent bone cement distribution patterns. **b** Separated bone cement distribution patterns

**Fig. 2:**
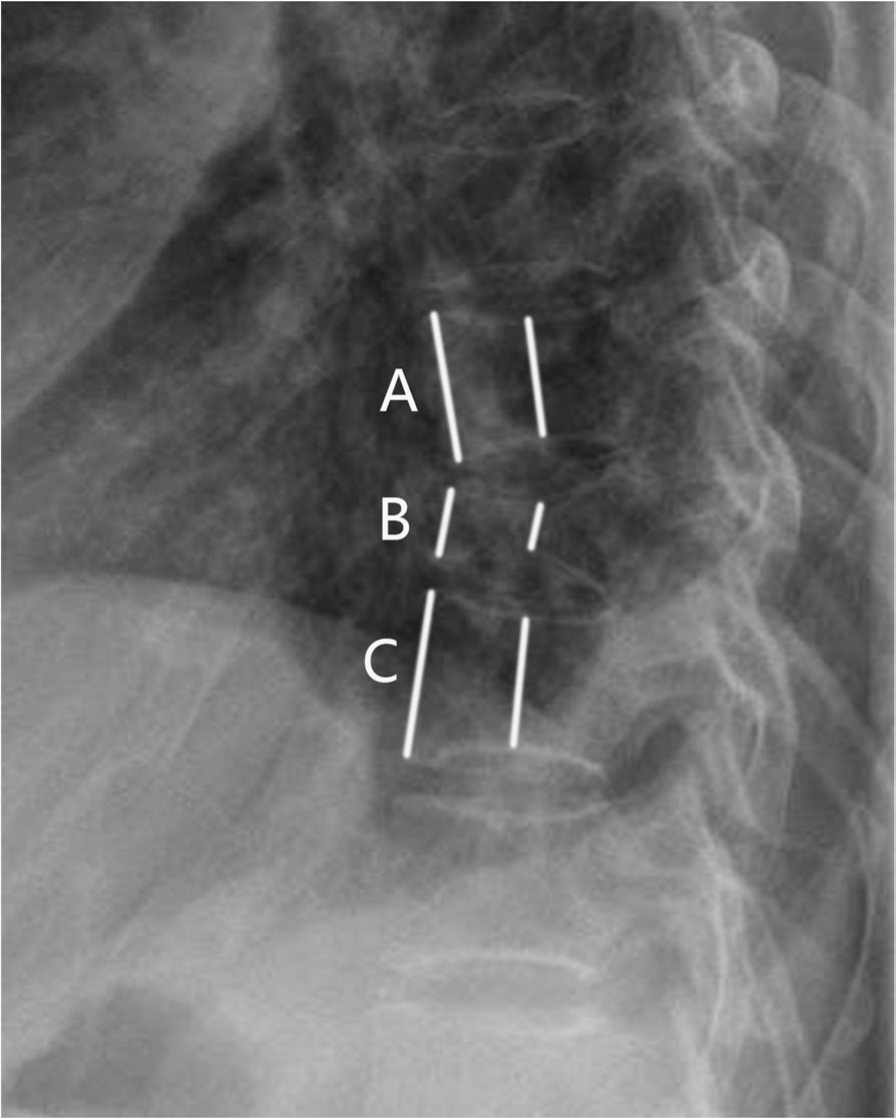
Measurement of vertebral compression ratio is done using the following formula: B/[(A + C)/2]. **A** Anterior vertebral height of upper vertebra, **B** anterior vertebral height of fracture level, **C** anterior vertebral height of lower vertebra

Informed consent was waived for this retrospective study. A total of 59 patients with single-level metastatic vertebral lesions who underwent bilateral PKP in our hospital from October 2016 to December 2019 were retrospectively reviewed. According to the different bone cement distribution patterns observed on frontal radiographs, patients were divided into confluent (*n* = 35, CF) or separated (*n* = 24, SP) groups. Confluent group: cement masses were uniformly spongy dispersed (Fig. 1a). Separated group: the bilateral bone cement masses were two separate masses with no or only a small part of contact between them (Fig. 1b). Through CT scans, osteolytic changes show non-consecutive, worm-eaten, hollow trabecular bone or vertebral walls. In contrast, osteoblast-related changes may correspond to nodular deposits, mottled deposits, or diffuse deposits [7]. Semi-fixed vertebrae (T3-T10, L5-S1), junctional vertebrae (T1, T2, T11-L1) and mobile vertebrae (L2-L4), as defined by the Spinal Instability Neoplastic Score (SINS). Pain was rated using visual analogue scale (VAS) scores (0: no pain; 1–3: mild pain; 4–6: moderate pain; and 7–10: severe pain), and neurological status was evaluated according to the Oswestry Disability Index (ODI, 0% indicates normal functional ability; 100% indicates severe spinal mobility dysfunction). The assessment of VAS and ODI principally followed up in the clinic, for patients inconvenient to come to the hospital was conducted via telephone questionnaire or via the Internet. Vertebral body height (VBH) variation was measured via lateral radiographs as follows: (fractured VBH/normal VBH)*100% (Fig. 2) [8]. VAS, ODI and VBH were assessed before intervention and 3 days, 1 month, 3 months and 1 year after intervention. The Medical Outcomes Study 36-item short form health survey (SF-36) used to measure quality of life (QoL) [9]. QoL scores were assessed before intervention and at 1 year after intervention. Preoperative longitudinal and cross-sectional imaging of each treated vertebra was evaluated to determine whether the tumour involved the posterior vertebral body and/or the pedicle and to quantify the percentage of bone invasion by the tumour. Postprocedure CT was performed to assess the bone cement distribution of each treated vertebra and to determine the presence of bone cement leakage. Indicators including operative duration and cement volume were compared between the two groups. Complications were classified as major or minor according to the Society of Interventional Radiology (SIR) reporting criteria [10].

All procedures were performed by the same experienced orthopaedist with 20 years of experience in spinal intervention. All patients were placed in the prone position under local anaesthesia (1% lidocaine; AstraZeneca, UK), with conscious sedation as needed. (13-gauge/2.3 mm for thoracic vertebrae and 11-gauge/2.9 mm for lumbar vertebrae, Sterylab, Italy) were inserted and driven through the pedicle to progress into the anterior one-third of the vertebral body under C-arm fluoroscopic guidance (Siemens Healthcare, Munich, Germany). High-viscosity barium-fortified polymethyl methacrylate (PMMA) bone cement (Weigao Medical GmbH, China) was slowly and carefully injected into the vertebral body after restoring the height of the vertebral body by intravertebral balloon inflation (Weigao Medical GmbH, China). Cement injection was terminated immediately when the cement reached the posterior one-fifth of the vertebral body or leakage was observed. After hardening the cement, the needles were removed, and the wound was disinfected and dressed with a sterile dressing. The bone cement volume and operative duration were recorded. All procedures were carried out under sterile conditions with strict adherence to aseptic practices. Postoperative plain radiographs and CT examinations were performed to assess the cement distribution and to check for cement leakage.

### Statistical analysis

Variables were first tested for normality using the Kolmogorov–Smirnov criterion. All continuous data are presented as the mean ± standard deviation (SD). Qualitative variables are expressed as absolute and relative frequencies. All data were statistically analyzed using SPSS 21.0 software (IBM Corp., Armonk, NY, USA). Pre- and postoperative comparisons of the continuous parameters were carried out by a paired-sample t-test. Comparisons of continuous parameters and score data between the UPK and BPK groups were carried out with the independent-sample t-test and Mann–Whitney U test, respectively. For non-normally distributed variables, a two sample nonparametric test was employed. The Chi-square test was used to compare proportions. A *p* value < 0.05 was considered to indicate a statistically significant difference.

## Results

### Demographic data

There were 19 (54.3%) males and 16 (45.7%) females in the CF group with an average age of 60.6 ± 9.7 years old and 13 (54.2%) males and 11 (45.8%) females in the SP group with an average age of 59.3 ± 10.0 years old. The numbers of affected thoracic and lumbar vertebrae were 13 and 22 in the CF group and 8 and 16 in the SP group, respectively. The CF group included 23 osteolytic lesions and 12 osteoblastic-related lesions, while the SP group included 18 osteolytic lesions and 6 osteoblastic-related lesions. With regard to the location of lesions, involvement of semi-fixed vertebrae, junctional vertebrae, and mobile vertebrae was noted in 17 cases, 11 cases, and 7 cases in the CF group, respectively. While involvement of semi-fixed vertebrae, junctional vertebrae and mobile vertebrae was noted in 5 cases, 11 cases, and 8 cases in the SP group, respectively. Posterior vertebral body and/or pedicle involvement was 74.3% (26/35) in the CF group and 66.7% (16/24) in the SP group. There were 11 vertebrae (11/35, 31.4%) with tumours involving 75% of the vertebral body volume in the CF group, with 5 vertebrae (5/25, 20.0%) with tumours involving 75% of the vertebral body volume in the SP group (Table 1). There were no significant differences in terms of age, sex, types of lesions, locations of lesions, posterior vertebral body and/or pedicle involvement, or percentage of vertebral invasion between the CF and SP groups (*p* > 0.05, Table 1). The average procedure duration was 75.1 ± 22.8 min in the CF group, which was similar to the procedure duration in the SP group (73.5 ± 33.6 min; *p* = 0.86). The average amount of cement injected was 3.8 ± 1.6 ml in the CF group, which was not significantly different from the amount injected in the SP group (3.9 ± 1.2 ml; *p* = 0.96). The average follow-up period was 33.1 ± 15.3 months (range 3–59 months) in the CF group and 26.3 ± 15.0 months (range 3–54 months) in the SP group (*p* = 0.97, Table 1).

**Table 1:** Demographic data of the 59 patients

	CF group	SP group	p
Number of patients	35	24	
Age(years)	60.6 ± 9.7	59.3 ± 10.0	0.62
Gender			
Male	19	13	1.00
Female	16	11	
Types of lesions			
Osteolytic	23	18	0.57
Osteoblastic-related	12	6	
Locations of lesions			
T3-T10, L5-S1	17	5	0.06
T11-L1	11	11	
L2-L4	7	8	
Posterior vertebral body and/or pedicle involvement			
Yes	26	16	0.53
None	9	8	
The percentage of vertebra invasion			
≤ 75%	11	5	0.32
> 75%	24	20	
Procedure duration(min)	75.1 ± 22.8	73.5 ± 33.6	0.86
Cement volume(mL)	3.8 ± 1.6	3.9 ± 1.2	0.96
Follow-up(months)	33.1 ± 15.3	26.3 ± 15.0	0.10

p: analyzed by independent-sample t-test or Chi-square test, CF group compared with SP group

*CF* Confluent, *SP* Separated

### Clinical and radiologic parameters

The recorded clinical and radiologic data recorded are shown in Table 2. No significant differences were observed in the preoperative VAS scores between the two groups (*p* > 0.05, Table 2). Pain was significantly alleviated after PKP compared with that before PKP at each follow-up point (*p* < 0.05, Table 2). Patients in the CF group achieved a more significant level of pain relief than did those in the SP group at 3 days and 1 month after PKP (*p* < 0.05, Table 2). There were no significant differences between the two groups in VAS scores 3 months and 1 year after PKP.

**Table 2:** Clinical and Radiologic Parameters after PKP

	CF group	SP group	p
VAS (score)			
Preoperative	6.4 ± 1.8	6.3 ± 2.0	0.78
3 days after PKP	2.8 ± 1.0*	3.7 ± 1.5*	0.01&
1 month after PKP	2.3 ± 0.6*	2.8 ± 0.9*	0.02&
3 months after PKP	3.1 ± 1.1*	3.0 ± 1.3*	0.75
1 year after PKP	3.6 ± 1.1*	3.7 ± 1.0*	0.92
ODI (score)			
Preoperative	71.2 ± 6.8	71.8 ± 6.3	0.74
3 days after PKP	30.6 ± 4.2*	31.6 ± 4.8*	0.42
1 month after PKP	30.5 ± 3.9*	31.0 ± 3.8*	0.62
3 months after PKP	31.2 ± 3.6*	30.7 ± 3.4*	0.57
1 year after PKP	30.8 ± 3.3*	31.0 ± 3.3*	0.83
Anterior-VBH variation(%)			
Preoperative	61.8 ± 5.9	60.9 ± 7.4	0.61
3 days after PKP	69.0 ± 6.5*	70.4 ± 7.3*	0.45
1 month after PKP	68.4 ± 6.4*	70.5 ± 6.7*	0.25
3 months after PKP	68.6 ± 6.3*	69.0 ± 5.9*	0.83
1 year after PKP	68.0 ± 6.1*	68.4 ± 6.4*	0.85
Middle-VBH variation(%)			
Preoperative	61.0 ± 5.6	60.6 ± 6.9	0.81
3 days after PKP	69.7 ± 5.9*	70.3 ± 7.1*	0.72
1 month after PKP	68.8 ± 5.7*	69.6 ± 7.0*	0.62
3 months after PKP	67.9 ± 5.6*	68.1 ± 6.2*	0.91
1 year after PKP	67.1 ± 5.7*	67.5 ± 6.1*	0.81
QoL(score)			
Preoperative	89.0 ± 16.5	93.7 ± 11.8	0.23
1 year after PKP	96.3 ± 18.5*	97.6 ± 19.2*	0.80

p: Analyzed by Mann–Whitney U test, the CF group compared with the SP group and Significant difference at *p* < 0.05

*CF* Confluent, *SP* Separated, *PKP* Percutaneous kyphoplasty, *VAS* Visual analog scale, *ODI* Oswestry disability index, *VBH* Vertebral body height, *QoL* Quality of life

^*^Significant difference at *p* < 0.05 compared with preoperation

There was no significant difference in the preoperative ODI scores between the two groups (*p* > 0.05, Table 2). The ODI scores of patients in the two groups decreased significantly after PKP (*p* < 0.05, Table 2). However, no significant difference was recorded between the two groups at any of the study time points during follow-up (*p* > 0.05, Table 2).

No significant difference was noted in the preoperative anterior VBH between the two groups (*p* > 0.05, Table 2). The postoperative anterior VBH of the two groups was significantly higher than that before PKP (*p* < 0.05, Table 2). There was no significant difference in the mean anterior VBH between the CF and SP groups at each study time point during follow-up (*p* > 0.05, Table 2). Similar results were found to middle-VBH variation (Table 2).

There was no significant difference in the preoperative QoL scores between the groups (*p* > 0.05, Table 2). The QoL scores of patients in the two groups increased significantly after PKP (*p* < 0.05, Table 2). However, no significant difference was recorded between the two groups at any of the study time points during follow-up (*p* > 0.05, Table 2).

**Fig. 3:**
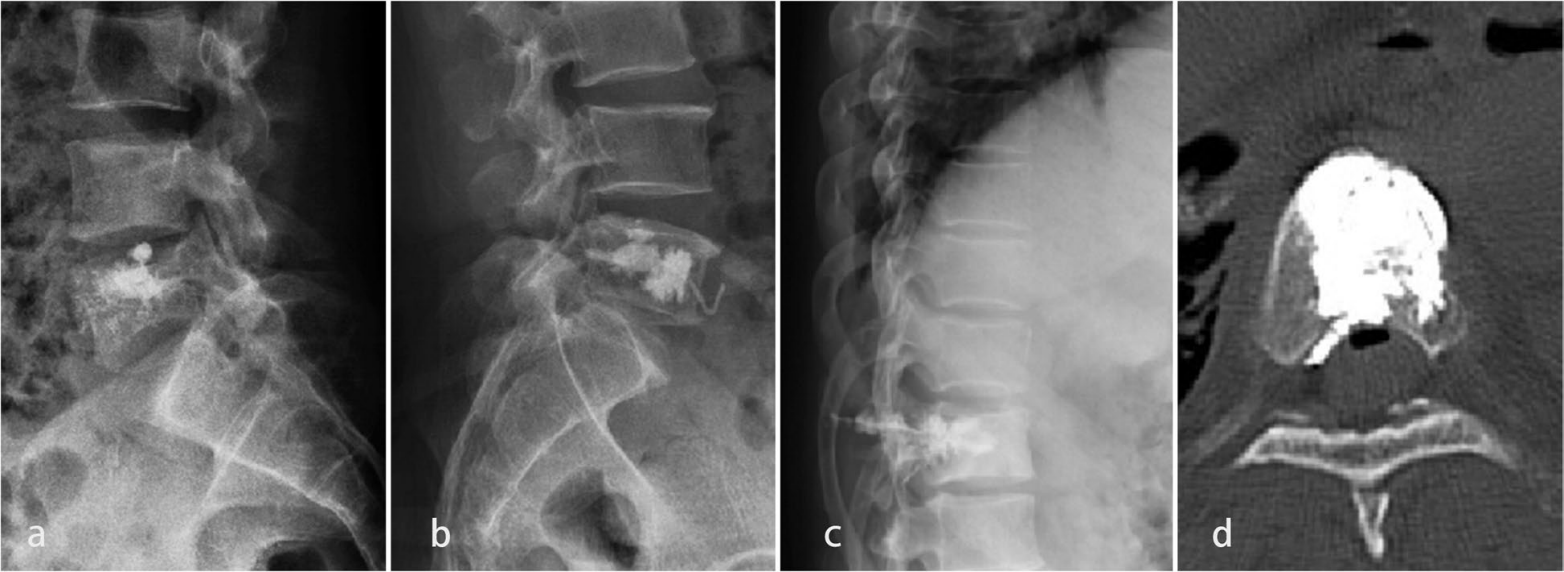
The types of cement leakage. Intervertebral leakage (**a**). Venous plexus leakage (**b**). Puncture trajectory (pedicle) leakage (**c**). Paraspinal tissue leakage (**d**)

Eight patients (22.9%) in the CF group suffered from bone cement leakage, including intervertebral leakage in 3 patients, basivertebral foramen leakage in 2 patients, puncture trajectory (pedicle) leakage in 2 patients, and venous plexus leakage in 1 patient (Fig. 3). Three patients (12.5%) in the SP group suffered from bone cement leakage, including intervertebral leakage in 1 patient, puncture trajectory (pedicle) leakage in 1 patient, and venous plexus leakage in 1 patient. Although the CF group had more leakage, the incidence of bone cement leakage in the CF group was not significantly higher than that in the SP group (*p* = 0.72, Table 3). None of the patients had experienced shock or sudden death due to bone cement implantation syndrome.

**Table 3:** Complications and follow-up results in the two groups

	CF group	SP group	p
Cement leakage			
Intervertebral disc	3	1	0.72
Basivertebral foramen	2	0	
Puncture trajectory (pedicle)	2	1	
Venous plexus	1	1	
Follow-up (3 months)			
Death	1	2	0.35
Survival	34	22	
Follow-up (1 year)			
Death	5	6	0.30
Survival	30	18	

p: analyzed by Chi-square test, CF group compared with SP group

*CF* Confluent, *SP* Separated

After treatment, the survival rates at 3 months and 1 year were 97.1% and 85.7% in the CF group, respectively. The survival rates at 3 months and 1 year were 91.7% and 75.0% in the SP group, respectively. No significant difference was found between the two groups in survival rate at 3 months and 1 year (*p* > 0.05, Table 3).

## Discussion

PKP for patients with metastatic vertebral lesions is usually palliative and focuses on reducing pain, improving function, restoring vertebral strength and improving quality of life [11]. Cement volume has been confirmed to be one of many factors markedly affecting clinical efficacy [12]. Previous studies have shown that cement with an extensive distribution of small volumes has the same effect as cement with a limited distribution of large volumes in OVCFs [5]. Furthermore, most relevant studies have focused on the outcomes of the different distribution patterns of cement in OVCFs, [2, 4–6] and the involvement of bone cement distribution patterns in metastatic vertebral lesions has not yet been well reported. Therefore, the present study aims to investigate the influence of diverse bone cement distribution patterns for patients with metastatic vertebral lesions after bilateral PKP.

Unlike OVCFs, which usually result in loss of VBH in the anterior part of the vertebral body, vertebral metastases (approximately 95%) usually invade the posterior part of the vertebral body and pedicles [13]. The extent of tumour involvement in the posterior vertebral body and/or pedicles and the percentage of vertebral invasion by tumours are important factors affecting the symptom palliation effect. No significant difference was observed in terms of the posterior vertebral body and/or pedicle involvement and the percentage of vertebral invasion between the two groups in the present study. Moreover, there was no significant difference in the location of lesions potentially affecting SINS scores between groups.

In terms of clinical efficacy, the CF group showed better pain relief than the SP group in terms of pain relief during the short-term follow-up (within one month), which is consistent with a previous study [5]. According to our experience, this could be explained by the following reasons. First, when the bilateral cement masses separated, the damaged trabeculae in the middle, which were not covered, still stimulated nerve endings. Second, the asymmetric distribution of bone cement may lead to instability of vertebrae under pressure load, and subsequent trabecular fretting. There was no significant difference between the CF group and the SP group on long-term follow-up (3 months and more), which may be related to the occurrence of new vertebral metastatic lesions and adjacent fractures associated with bone cement leakage, such as intervertebral leakage. Additionally, patients from both groups experienced substantial improvement in terms of ODI, VBH, and QoL after PKP in the present study, irrespective of the bone cement distribution pattern, which is consistent with previous study [8]. More importantly, patients with spinal metastases will develop new metastatic vertebral lesions, resulting in pain and deterioration in function and quality of life over time. Nevertheless, there was a sustained improvement in the VAS score, ODI and QoL in both groups in the present study. This might be related to the fact that these patients were treated with additional therapies after PKP, such as radiotherapy (RT), which might have contributed to local tumour control. Vertebral augmentation in combination with postoperative RT has been proven to be a good treatment strategy for spinal metastases [14]. While postoperative RT acted as a confounder, there is no denying that the management of spinal metastases requires multidisciplinary input.

In terms of clinical safety, cement leakage is the most common complication of PKP and is related to cement distribution [15, 16]. Although previous studies have shown that bone cement leakage is higher in dispersed cement than in dispersed cement [2], there was no difference between the two groups in the present study. The rate of overall leakage was comparatively lower than previously published results; moreover, most cases were asymptomatic and did not require further treatment [17]. The lower rate of bone cement leakage could be explained by the following reasons. First, we prefer to use high-viscosity bone cement, which could significantly reduce the incidence rate of cement leakage compared to low-viscosity bone cement [18]. Second, the application of barium sulfate to achieve proper cement turbidity increased the visibility of the cement and allowed early detection, while there was only slight cement leakage. Third, the use of high-resolution fluoroscopy contributed to the early detection of small leaks. Fourth, when PMMA became mushy and sticky enough to not drip off the bone cement inserter, it was injected into the cavity to reduce the possibility of leakage. The destruction of the vertebral wall is considered a contraindication for vertebral augmentation [19]. On the basis of observations and for practical purposes, we prefer to wait approximately 1–2 min for the cement to become viscous after the first injection which acts as an anchor for the next injection on the same side of vertebra.

Although we attempted to position the access needle tip at or beyond the midline to facilitate pedicle-to-pedicle and endplate-to-endplate bone cement filling in clinical practice, the expected bone cement distribution may not be achieved in many patients. The different distribution of bone cement in PKP may be related to many factors. First, high-viscosity bone cement could significantly achieve a better spread homogeneous in the body of the vertebra compared to low-viscosity bone cement [20]. Second, a more lateral puncture point with a greater camber angle may connect bilateral bone cement in the middle of the vertebral body [21]. Third, temperature differences (verbal body temperature and operating room temperature) may affect the spread homogeneous distribution of bone cement; the higher the temperature, the faster the solidification [22]. Fourth, vertebrae can be filled with hyperplastic and metastatic tissues by a variety of tumours, which can hinder the homogeneous diffusion of bone cement [23]. However, because these factors are not well documented and preserved, retrospective measurements and comparisons between the two groups are impossible, which will be the subject of future studies. Although the effectiveness of radiofrequency ablation (RFA) in spinal metastases has been reported in many studies, some hold different opinions that RFA does not offer any clear added benefit combined with vertebral augmentation [24], which is why thermal ablation was not performed in the present study and why assessment of the distribution of cement following ablation was not evaluated. Overall, the differences in clinical influence including efficacy and safety, between the two different types of cement distribution are of negligible value. Moreover, although the operator tries to produce a certain type of cement distribution after vertebral augmentation, an expected distribution may not be achieved in many patients, particularly patients with pathologic fractures. Therefore, there is no need to try to produce a certain type of cement distribution pattern with more needle manipulation that potentially increases the complication rate.

The present study is not without limitations. First, this study was a single-centre, retrospective study rather than a prospective study, which exhibited selection bias. Second, we limited the analysis to patients with a single-segment lesion to create a uniform cohort, simplify interpretation of the results and eliminate some study confounders, resulting in a relatively limited number of patients since most patients are referred from other cancer centres because of severe multiple-segment spinal metastases. More importantly, we were unable to accurately collect complete data about previous treatment history (including radiotherapy, analgesics) and bone mineral density. Third, the VAS used in the present study was not specifically applied to assess pain caused by vertebral metastasis, as pain may be caused by other sites of metastasis. Furthermore, pain medication usage, which could potentially confound VAS was not well documented. Fourth, another limitation is that the primary cancers of both groups were different, while various primary malignancies with different prognoses could have affected the VAS, ODI and QoL scores.

## Conclusions

More rapid pain relief was gained with confluent rather than separated bone cement distribution patterns in PKP for patients with metastatic vertebral lesions.

## Data Availability

All data generated or analyzed in this study are included in the article.
